# Association between diabetes mellitus and cause of death in patients with tuberculosis: A Korean nationwide cohort study

**DOI:** 10.1371/journal.pone.0295556

**Published:** 2023-12-14

**Authors:** Se Hyun Kwak, Dawoon Jeong, Jeongha Mok, Doosoo Jeon, Hee-Yeon Kang, Hee Jin Kim, Hee-Sun Kim, Hongjo Choi, Young Ae Kang

**Affiliations:** 1 Division of Pulmonology, Allergy and Critical Care Medicine, Department of Internal Medicine, Yongin Severance Hospital, Yonsei University College of Medicine, Yongin, Republic of Korea; 2 Department of Preventive Medicine, Seoul National University, College of Medicine, Seoul, Republic of Korea; 3 Department of Internal Medicine, Pusan National University Hospital, Pusan National University School of Medicine, Busan, Republic of Korea; 4 Department of Internal Medicine, Pusan National University Yangsan Hospital, Pusan National University School of Medicine, Yangsan, Republic of Korea; 5 Department of Cancer Control and Population Health, National Cancer Center Graduate School of Cancer Science and Policy, Goyang, Republic of Korea; 6 Jeju double cross clinic, Korean National Tuberculosis Association, Jeju, Republic of Korea; 7 Department of Health Policy Research, National Evidence-Based Healthcare Collaborating Agency, Seoul, Republic of Korea; 8 Department of Preventive Medicine, Konyang University College of Medicine, Daejeon, Republic of Korea; 9 Division of Pulmonary and Critical Care Medicine, Department of Internal Medicine, Severance Hospital, Yonsei University College of Medicine, Seoul, Republic of Korea; 10 Institute for Immunology and Immunological Diseases, Yonsei University College of Medicine, Seoul, Republic of Korea; The University of Georgia, UNITED STATES

## Abstract

Despite its significant impact on mortality, tuberculosis (TB)-diabetes mellitus (DM) co-prevalence has not been well-elucidated for the cause of death. We investigated the impact of DM on TB-related and non-TB-related deaths in patients with TB. This retrospective nationwide cohort study included patients diagnosed with TB between 2011 and 2017 in South Korea. We performed Fine and Gray regression model analyses to assess the mortality risk of DM classified by cause of death. Of 239,848 patients, 62,435 (26.0%) had DM, and 20,203 died during anti-TB treatment. Of all deaths, 47.9% (9,668) were caused by TB, and the remaining 52.1% (10,535) was attributed to various non-TB-related causes. The mortality rate was higher in the DM than in the non-DM groups in both men and women. DM was associated with a higher risk of TB-related (adjusted hazard ratio [aHR] 1.07, 95% confidence interval [CI] 1.01–1.13) and non-TB-related (aHR 1.21, 95% CI 1.15–1.27) deaths in men; however, only a higher risk of non-TB-related deaths (aHR 1.29, 95% CI 1.20–1.38) in women. Our findings indicate that DM is independently associated with a greater risk of death during anti-TB treatment among patients with TB for both TB-related and non-TB-related deaths.

## Introduction

Tuberculosis (TB) is the leading cause of morbidity and mortality from a single epidemic worldwide, despite advances in diagnosis and treatment [1]. Among the members of the Organization for Economic Cooperation and Development, South Korea has a relatively high TB-related mortality rate (4 per 100,000 individuals), despite its high-income status and the implementation of a national strategy for TB prevention and care [2]. This high mortality rate may be attributed to co-morbid non-communicable diseases (NCDs). With economic growth, the burden of NCD has increased over that of other communicable diseases in many countries [3]. There is growing evidence of an association between TB and NCD, including diabetes mellitus (DM), cardiovascular diseases, and cancer [4, 5]. NCD may delay TB diagnosis or worsen the patient’s general condition, negatively affecting treatment adherence and increasing the risk of treatment failure and death [5].

In South Korea, DM is highly prevalent in adults aged 19 years or older (13.9% in 2020) [6]. Particularly, the prevalence of DM in the older population (>65 years) is 27.6% [7]. Among individuals newly discovered to have TB in 2021 in South Korea, the number of older patients was also high (51%) [8]. Additionally, the prevalence of DM among patients with TB was 26.8%, and it increased with age, reaching 40% in older patients (>65 years) with TB [9]. Understanding the impact of DM on TB outcomes and strengthening the national strategy for managing patients with TB and DM is a critical public health issue in Korea.

Several studies have reported the impact of DM on TB treatment outcomes [10]. Patients with DM have higher mortality rates and a greater risk of TB recurrence post-treatment is observed in patients with DM than in those without DM [11–18]. However, the precise biological or socio-behavioral mechanism underlying how DM increases the risk of death in TB patients remains unclear. Moreover, few studies have examined the impact of DM on TB-related and non-TB-related mortality in patients with TB. In this study, we investigated the impact of DM on TB-related and non-TB-related mortality in patients with TB using integrated data from a national TB cohort.

## Methods

### Study design and setting

This study was a retrospective nationwide cohort study of patients with TB. We used the Korean Tuberculosis and Post-Tuberculosis cohort, which was constructed by linking the following three databases [19]: 1) the Korean National Tuberculosis Surveillance System (KNTSS); 2) the National Health Insurance Database (NHID); and 3) Statistics Korea data on the causes of death in patients with TB registered between 2011 and 2018.

### Study participants

Patients diagnosed with TB between 2011 and 2018 were considered eligible. The primary exposure variable was DM. The study population was classified into DM and non-DM groups. DM was defined as the identification of any of the following criteria for 1 year before and after TB diagnosis: 1) at least two claims of International Classification of Diseases (ICD) codes for DM (E11-E14), and 2) at least one claim of ICD codes for DM and the prescription of anti-diabetic drugs for more than 4 weeks.

**Fig 1:**
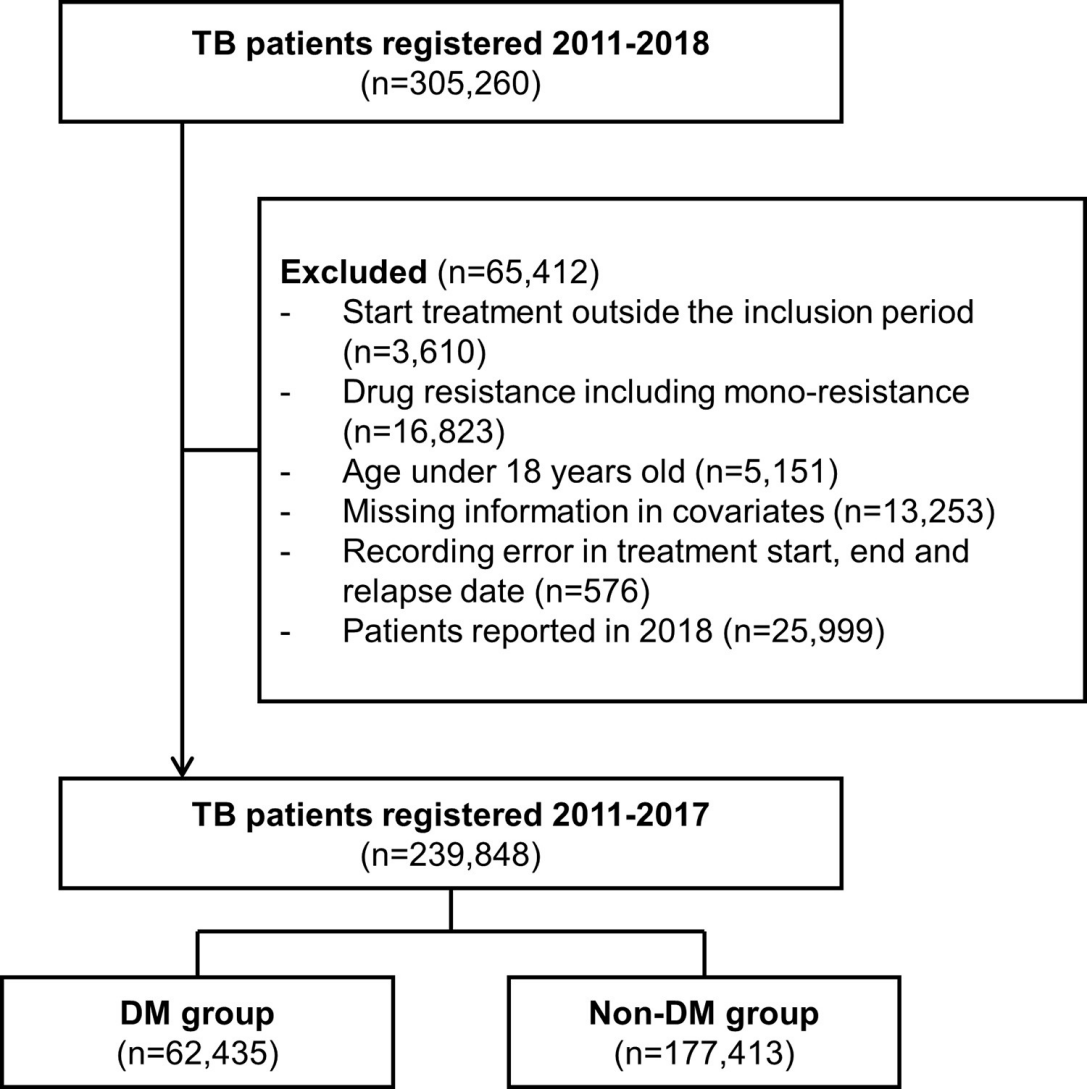
Flow diagram of the study population. Abbreviations: TB, Tuberculosis; DM, diabetes mellitus.

Initially, 305,260 patients were identified by integrating the KNTSS and NHID between 2011 and 2018. Among these patients, we selected our subset study population to patients with drug-susceptible TB between 2011 and 2017 to analyze the treatment outcome. From the main integrated 305,260 patients, we excluded those who started receiving treatment outside the inclusion period (n = 3,610), those with drug resistance (n = 16,823), those aged under 18 years (n = 5,151), those with missing information on covariates (n = 13,253), those with documentation errors regarding treatment start and end dates (n = 576), and those reported in 2018 (n = 25,999). Finally, 239,848 patients were included in the final analysis. Of the included patients, 62,435 (26.0%) and 177,413 (74.0%) belonged to the DM and non-DM groups, respectively (Fig 1).

### Covariates

The following variables were measured as covariates that might influence the final treatment outcome: sex; age; household income; TB lesions; previous TB history; results of sputum acid-fast bacillus (AFB) smear and culture; presence of co-morbidities (transplantation, human immunodeficiency virus, end-stage renal disease [ESRD], and cancer); and Charlson co-morbidity index (CCI) score. CCI was calculated as previously described [20]. The CCI is a method of classifying comorbidities of patients based on the ICD Codes. ICD-10 codes were investigated within 1 year preceding the index date of TB for calculating CCI score. The household income of health insurance beneficiaries was classified to the 5th quintile (1 = lowest, 5 = highest), according to the national health insurance premium, and medical aid beneficiaries were classified into group 0.

### Treatment outcomes

Treatment outcomes were defined according to the criteria suggested by the World Health Organization [21] and reported to the KNTSS. Treatment success was defined as the sum of cure and treatment completion. Death was defined as any mortality during treatment. Classification of death into TB-related and non-TB-related deaths was based on Statistics Korea data on the causes of death and KNTSS outcomes regarding the cause of death.

### Statistical analysis

Continuous variables are presented as mean (standard deviation) for normally distributed variables and median (interquartile range, IQR) for non-normally distributed variables. Categorical variables are expressed as numbers (percentages). The Student’s t-test or Mann–Whitney U test was used to compare continuous variables, and the chi-square test was used to compare categorical variables.

To account for the competing risk of the cause of death (TB-related and non-TB-related), the mortality risk of DM was estimated using the Fine and Gray regression model by calculating the sub-distribution hazard ratios with a 95% confidence interval (CI). The cumulative mortality rates between the DM and non-DM groups were compared using Gray’s test.

All p-values were two-tailed, and a p-value of < 0.05 was considered statistically significant for all analyses. All statistical analyses were performed using SAS Enterprise Guide (SAS Institute Inc., Cary, NC, USA) and STATA/MP version 17 (Stata Corp LLC, College Station, TX, USA).

### Ethical approval

The study protocol was reviewed and approved by the Institutional Review Board of the National Evidence-Based Healthcare Collaborating Agency (NECAIRB19-008-1).

## Results

### Baseline characteristics of patients with TB classified by DM status

Table 1 shows the baseline characteristics of patients with TB according to their DM status. Among the 239,848 participants, 140,939 (58.8%) were men with a median age of 56 years. There were more men in the DM group than in the non-DM group (64.8% vs. 56.6%, p<0.001), and the median age of the DM group was higher than that of the non-DM group (65 years vs. 51 years, p <0.001). Regarding household income, more patients in the DM group than that in the non-DM group belonged to the lowest income group (12.2% vs. 6.5%, p<0.001). More patients in the DM group had positive AFB smear (35.5% vs. 27.0%, p<0.001) and mycobacterial culture (47.3% vs. 40.8%, p<0.001) results than that in the non-DM group. Additionally, patients in the DM group had higher CCI scores and more co-morbidities, including organ transplantation, malignant disease, and ESRD, than those in the non-DM group (Table 1). These differences in characteristics between the DM and non-DM groups were also consistent between men and women.

**Table 1:** Baseline characteristics of the study participants.

Variables	Total (n = 239,848)			Men (n = 140,939)			Women (n = 98,909)		
	DM (n = 62,435)	Non-DM (n = 177,413)	*p*	DM (n = 40,446)	Non-DM (n = 100,493)	*p*	DM (n = 21,989)	Non-DM (n = 76,920)	*p*
Sex									
Men	40,446 (64.8)	100,493 (56.6)	<0.001						
Women	21,989 (35.2)	76,920 (43.4)							
Age, years, median (IQR)	65 (56–77)	51 (35–70)	<0.001	63 (53–74)	51 (36–67)	<0.001	74 (64–80)	51 (34–73)	<0.001
Age, years									
18–24	226 (0.4)	15,051 (8.5)	<0.001	131 (0.3)	8,317 (8.3)	<0.001	95 (0.4)	6,734 (8.8)	<0.001
25–34	893 (1.4)	27,917 (15.7)		550 (1.4)	14,624 (14.6)		343 (1.6)	13,293 (17.3)	
35–44	3,365 (5.4)	26,642 (15.0)		2,665 (6.6)	15,285 (15.2)		700 (3.2)	11,357 (14.8)	
45–54	9,584 (15.4)	28,741 (16.2)		7,935 (19.6)	18,200 (18.1)		1,649 (7.5)	10,541 (13.7)	
55–64	12,931 (20.7)	24,805 (14.0)		10,104 (25.0)	15,910 (15.8)		2,827 (12.9)	8,895 (11.6)	
65–74	15,085 (24.2)	21,991 (12.4)		9,522 (23.5)	13,231 (13.2)		5,563 (25.3)	8,760 (11.4)	
≥75	20,351 (32.6)	32,266 (18.2)		8,428 (23.6)	14,926 (14.9)		10,812 (49.2)	17,340 (22.5)	
Household income									
0 (Lowest)	7,595 (12.2)	11,540 (6.5)	<0.001	4,668 (11.5)	6,679 (6.6)	<0.001	2,927 (13.3)	4,861 (6.3)	<0.001
1	9,816 (15.7)	28,922 (16.3)		6,422 (15.9)	16,183 (16.1)		3,394 (15.4)	12,739 (16.6)	
2	8,594 (13.8)	30,150 (17.0)		6,050 (15.0)	17,512 (17.4)		2,544 (11.6)	12,638 (16.4)	
3	9,471 (15.2)	32,176 (18.1)		6,402 (15.8)	18,561 (18.5)		3,069 (14.0)	13,615 (17.7)	
4	11,268 (18.1)	34,184 (19.3)		7,458 (18.4)	19,533 (19.4)		3,810 (17.3)	14,651 (19.0)	
5 (Highest)	15,691 (25.1)	40,441 (22.8)		9,446 (23.4)	22,025 (21.9)		6,245 (28.4)	18,416 (23.9)	
TB Lesion									
Pulmonary	54,449 (87.2)	152,222 (85.8)	<0.001	36,235 (83.2)	88,718 (88.3)	<0.001	18,214 (82.8)	63,504 (82.6)	0.345
Extra-pulmonary	7,986 (12.8)	25,191 (14.2)		4,211 (10.4)	11,775 (11.7)		3,775 (17.2)	13,416 (17.4)	
Prior TB history	8,807 (14.1)	23,322 (13.2)	<0.001	6,811 (16.8)	15,618 (15.5)	<0.001	1,996 (9.1)	7,704 (10.1)	<0.001
AFB smear, positive	22,144 (35.5)	47,807 (27.0)	<0.001	15,076 (37.3)	28,160 (28.0)	<0.001	7,068 (32.1)	19,647 (25.5)	<0.001
TB Culture, positive	29,554 (47.3)	72,456 (40.8)	<0.001	19,875 (49.1)	42,864 (42.7)	<0.001	9,679 (44.0)	29,592 (38.5)	<0.001
Co-morbidities									
Cancer	2,465 (4.0)	3,890 (2.2)	<0.001	1,879 (4.6)	2,652 (2.6)	<0.001	586 (2.7)	1,238 (1.6)	<0.001
Transplantation	448 (0.7)	280 (0.2)	<0.001	319 (0.8)	192 (0.2)	<0.001	129 (0.6)	88 (0.1)	<0.001
HIV	96 (0.2)	237 (0.1)	0.240	85 (0.2)	218 (0.2)	0.800	11 (0.1)	19 (0.0)	0.060
ESRD	3,138 (5.0)	927 (0.5)	<0.001	1,969 (4.9)	546 (0.5)	<0.001	1,169 (5.3)	381 (0.5)	<0.001
CCI score									
0	18,374 (29.4)	82,558 (46.5)	<0.001	13,474 (33.3)	49,286 (49.0)	<0.001	4,900 (22.3)	33,272 (43.3)	<0.001
1	25,863 (41.4)	72,869 (41.1)		16,548 (40.9)	39,544 (39.4)		9,315 (42.4)	33,325 (43.3)	
2	4,806 (7.7)	6,915 (3.9)		2,866 (7.1)	3,746 (3.7)		1,940 (8.8)	3,169 (4.1)	
≥3	13,392 (21.5)	15,071 (8.5)		7,558 (18.7)	7,917 (7.9)		5,834 (26.5)	7,154 (9.3)	
Notification year									
2011	9,435 (15.1)	32,829 (18.5)	<0.001	6,280 (15.5)	18,790 (18.7)	<0.001	3,155 (14.3)	14,039 (18.3)	<0.001
2012	9,757 (15.6)	30,493 (17.2)		6,426 (15.9)	17,288 (17.2)		3,331 (15.1)	13,205 (17.2)	
2013	8,829 (14.1)	26,449 (14.9)		5,820 (14.4)	14,819 (14.7)		3,009 (13.7)	11,630 (15.1)	
2014	8,863 (14.2)	24,941 (14.1)		5,627 (13.9)	14,029 (14.0)		3,236 (14.7)	10,912 (14.2)	
2015	8,617 (13.8)	22,227 (12.5)		5,547 (13.7)	12,616 (12.6)		3,070 (14.0)	9,611 (12.5)	
2016	8,612 (13.8)	21,216 (12.0)		5,503 (13.6)	12,074 (12.0)		3,109 (14.1)	9,142 (11.9)	
2017	8,322 (13.3)	19,258 (10.9)		5,243 (13.0)	10,877 (10.8)		3,079 (14.0)	8,381 (10.9)	

**Note:** Data are presented as numbers (%) or median (IQR)

**Abbreviations:** DM, diabetes mellitus; TB, tuberculosis; AFB, acid-fast bacillus; HIV, human immunodeficiency virus; ESRD, end-stage renal disease; CCI, Charlson comorbidity index

### Treatment outcomes at the end of treatment in the DM and non-DM groups

Treatment outcomes were analyzed for all participants (Table 2). The treatment success rate was lower in the DM vs. non-DM group (76.8% vs. 85.5%, p<0.001), and mortality rate was higher in the DM vs. non-DM group (14.6% vs. 6.3%, p<0.001) in both men (14.4% vs. 7.0%, p<0.001) and women (6.9% vs. 2.9%, p<0.001). TB-related and non-TB-related deaths accounted for 4.0% and 4.4%, respectively in total participants. TB-related death occurred in 6.4% and 3.2% of patients in the DM and non-DM groups, respectively (p<0.001). Non-TB-related deaths were reported in 8.1% and 3.1% of patients in the DM and non-DM groups, respectively (p<0.001). Both TB-related and non-TB-related deaths were more predominant in the DM group than in the non-DM group.

**Table 2:** Tuberculous treatment outcomes at the end of treatment by DM status.

Treatment outcomes at the EOT	Total (n = 239,848)			Men (n = 140,939)			Women (n = 98,909)		
	DM (n = 62,435)	Non-DM (n = 177,413)	*p*	DM (n = 40,446)	Non-DM (n = 100,493)	*p*	DM (n = 21,989)	Non-DM (n = 76,920)	*p*
Treatment success	47,952 (76.8)	151,619 (85.5)	<0.001	30,874 (76.3)	84,042 (83.6)	<0.001	17,078 (77.7)	67,577 (87.9)	<0.001
Treatment failure	53 (0.1)	104 (0.1)	0.207	46 (0.1)	82 (0.1)	0.7	7 (0.0)	22 (0.0)	0.804
Death during treatment	9,083 (14.6)	11,120 (6.3)	<0.001	5,833 (14.4)	6,985 (7.0)	<0.001	3,250 (14.8)	4,135 (5.4)	<0.001
TB-related death	4,010 (6.4)	5,658 (3.2)	<0.001	2,486 (6.2)	3,405 (3.4)	<0.001	1,524 (6.9)	2,253 (2.9)	<0.001
Non-TB-related death	5,073 (8.1)	5,462 (3.1)	<0.001	3,347 (8.3)	3,580 (3.6)	<0.001	1,726 (7.9)	1,882 (2.5)	<0.001
Lost to FU & Not evaluated	5,347 (8.6)	14,570 (8.2)	0.006	3,693 (9.1)	9,384 (9.3)	0.225	1,654 (7.5)	5,186 (6.7)	<0.001

**Note:** Data are presented as numbers (%) or median (IQR)

**Abbreviations:** EOT, end of treatment; DM, diabetes mellitus; TB, tuberculosis; FU, follow up

### Causes of death and timeline of TB-related and non-TB-related deaths

The median time between treatment initiation and death was 45 days (IQR 13–116 days). TB-related deaths occurred earlier than non-TB-related deaths, with a median time to death of 32 days (IQR 10–83) for TB-related death and 64 days (IQR 18–144 days) for non-TB-related death in the study population. S1 Fig shows the number of TB-related and non-TB-related deaths according to the time of treatment initiation in the DM (S1A Fig) and non-DM groups (S1B Fig). Of 20,203 deaths, 11,582 (57.3%) occurred during the initial 2-month intensive phase of anti-TB treatment. Of them, 6,494 (56.1%) were TB-related and 5,088 (43.9%) were non-TB-related. In the DM group, among 9,083 total deaths, 4,905 (54%) occurred in the initial 2 months (52.6% TB-related and 47.4% non-TB-related). Similarly, of the 11,120 deaths in the non-DM group, 6,677 (60%) occurred in the initial 2 months (58.6% TB related and 41.4% non-TB related).

TB was the most common cause of death (9,668 deaths, 47.9%). Among 10,535 non-TB-related deaths, lung cancer (n = 1,205, 11.4%) and pneumonia (n = 976, 9.3%) were common causes of death. The top 10 causes of non-TB-related death in the DM and non-DM groups are shown in S2 Fig. The top causes of non-TB-related deaths, including lung cancer, pneumonia, and cerebrovascular disease, were similar between the DM and non-DM groups. DM was the third leading cause of death in the DM group.

### Different unfavorable effects of DM on TB-related and non-TB-related deaths in men and women

**Fig 2:**
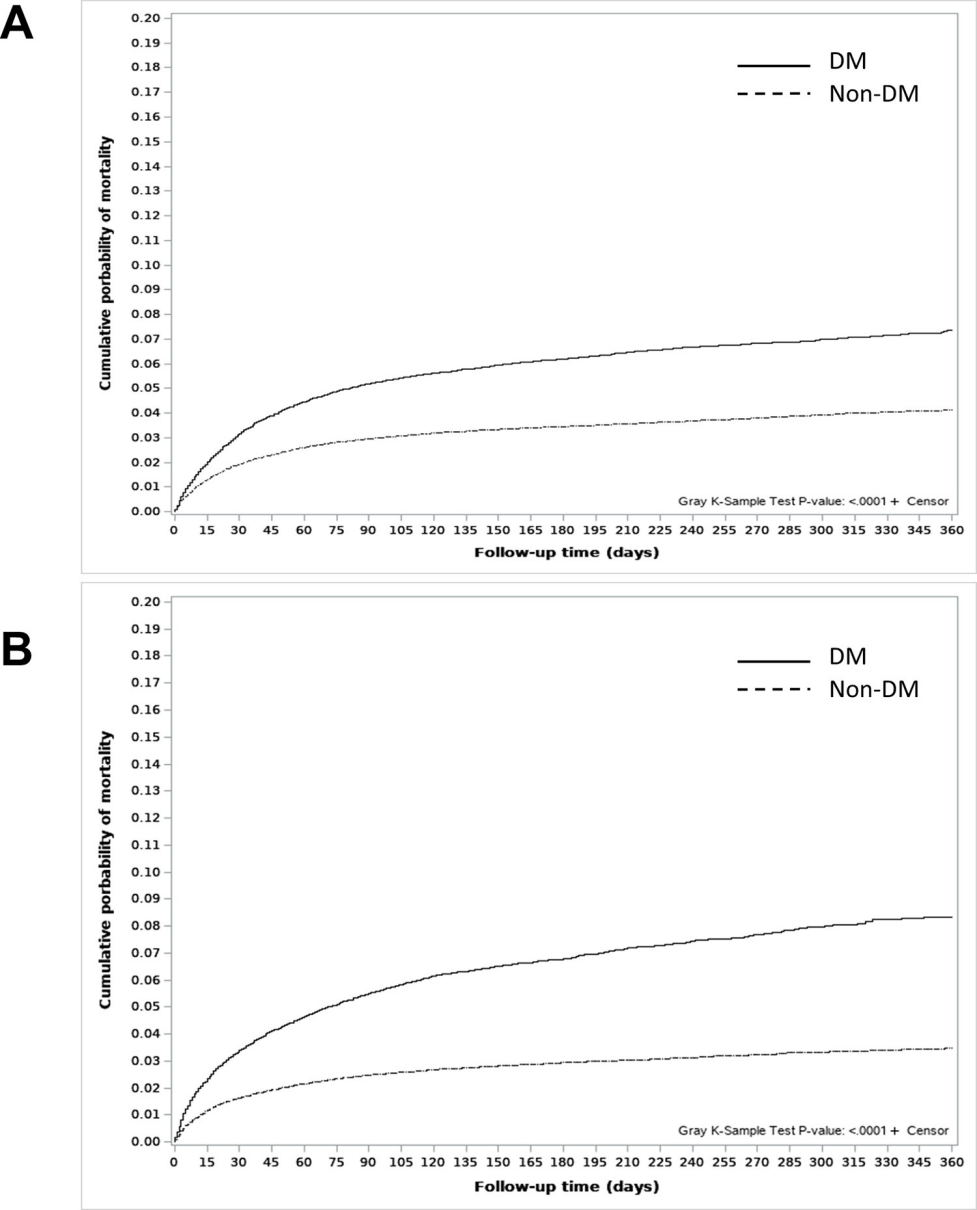
Cumulative mortality curves for TB-related deaths in men and women according to DM status. (A) Cumulative mortality curves for TB-related deaths in men. (B) Cumulative mortality curves for TB-related deaths in women. Abbreviations: TB, tuberculosis; DM, diabetes mellitus.

The cumulative mortality curves showed a higher probability of TB-related deaths in the DM group than in the non-DM group in both men and women (Fig 2, Gray’s test, p<0.001). However, in the multivariate-adjusted sub-distribution hazard model, the unfavorable impact of DM on TB-related death was consistent in men (adjusted hazard ratio, aHR 1.07, 95% CI 1.01–1.13) but not in women (aHR 1.06, 95% CI 0.99–1.13) (Table 3). Older age, lower household income, AFB smear positivity, and higher CCI scores were unfavorable prognostic factors for TB-related deaths in both men and women.

**Table 3:** Impact of DM on TB-related deaths in men and women.

Variables	Men (n = 140,939)			Women (n = 98,909)		
	HR	aHR	*p*	HR	aHR	*p*
DM	1.81 (1.72–1.90)	1.07 (1.01–1.13)	0.020	2.41 (2.26–2.57)	1.06 (0.99–1.13)	0.120
Age, years						
18–24	Ref	Ref		Ref	Ref	
25–34	3.12 (1.39–6.96)	2.91 (1.30–6.50)	0.001	2.47 (0.85–7.16)	2.50 (0.86–7.29)	0.090
35–44	11.10 (5.21–23.63)	9.07 (4.26–19.32)	<0.001	5.99 (2.17–16.51)	6.11 (2.20–16.97)	<0.001
45–54	25.48 (12.10–53.65)	18.10 (8.58–38.16)	<0.001	10.64 (3.94–28.79)	10.54 (3.86–28.75)	<0.001
55–64	32.82 (15.62–9.10)	23.45 (11.13–49.40)	<0.001	17.47 (6.52–46.82)	16.67 (6.17–45.10)	<0.001
65–74	58.30 (27.75–22.48)	41.78 (19.85–87.94)	<0.001	44.46 (16.77–117.84)	38.36 (14.33–102.71)	<0.001
≥ 75	175.69 (83.77–368.50)	113.94 (54.18–239.61)	<0.001	201.72 (76.47–532.09)	142.66 (53.48–380.52)	<0.001
Household income						
0 (Lowest)	1.61 (1.48–1.75)	1.75 (1.61–1.91)	<0.001	2.05 (1.86–2.26)	1.49 (1.35–1.65)	<0.001
1	0.81 (0.75–0.88)	1.33 (1.22–1.44)	<0.001	0.89 (0.80–0.98)	1.22 (1.10–1.35)	<0.001
2	0.66 (0.60–0.72)	1.29 (1.19–1.41)	<0.001	0.65 (0.58–0.73)	1.25 (1.12–1.41)	<0.001
3	0.65 (0.59–0.70)	1.15 (1.06–1.26)	0.001	0.65 (0.58–0.72)	1.15 (1.03–1.28)	0.020
4	0.71 (0.66–0.77)	1.05 (0.97–1.13)	0.280	0.75 (0.68–0.83)	1.11 (1.00–1.23)	0.040
5 (Highest)	Ref			Ref	Ref	
TB Lesion						
Pulmonary	Ref	Ref		Ref	Ref	
Extra-pulmonary	0.65 (0.59–0.71)	0.72 (0.64–0.81)	<0.001	0.656 (0.51–0.62)	0.62 (0.54–0.70)	<0.001
Prior TB history						
No	Ref	Ref		Ref	Ref	
Yes	1.29 (1.21–1.38)	1.07 (1.00–1.14)	0.050	0.95 (0.85–1.07)	1.08 (0.97–1.21)	0.160
AFB smear						
Negative	Ref	Ref		Ref	Ref	
Positive	2.80 (2.65–2.96)	2.50 (2.36–2.65)	<0.001	2.50 (2.33–2.68)	1.82 (1.68–1.96)	<0.001
Unknown	1.02 (0.92–1.12)	0.82 (0.73–0.93)	0.001	0.83 (0.75–0.92)	0.72 (0.63–0.83)	<0.001
TB culture						
Negative	Ref	Ref		Ref	Ref	
Positive	1.44 (1.34–1.54)	0.92 (0.86–0.99)	0.030	1.42 (1.30–1.55)	0.82 (0.74–0.89)	<0.001
Unknown	1.67 (1.55–1.80)	1.63 (1.50–1.77)	<0.001	1.42 (1.30–1.55)	1.67 (1.50–1.86)	<0.001
CCI score						
0	Ref	Ref		Ref	Ref	
1	1.78 (1.67–1.91)	1.00 (0.94–1.08)	0.900	1.40 (1.28–1.54)	0.70 (0.64–0.77)	<0.001
2	3.64 (3.29–4.02)	1.80 (1.63–2.00)	<0.001	5.29 (4.71–5.93)	1.74 (1.55–1.96)	<0.001
≥3	5.18 (4.83–5.56)	1.90 (1.76–2.05)	<0.001	6.15 (5.62–6.72)	1.54 (1.40–1.70)	<0.001
Co-morbidities						
Transplantation	0.67 (0.40–1.11)	0.88 (0.53–1.47)	0.630	0.82 (0.39–1.73)	2.02 (0.94–4.34)	0.070
HIV	1.17 (0.72–1.91)	1.42 (0.85–2.37)	0.180	2.90 (0.92–9.15)	3.06 (0.83–11.21)	0.090
Cancer	0.54 (0.45–0.66)	0.40 (0.33–0.49)	<0.001	0.32 (0.21–0.49)	0.41 (0.27–0.63)	<0.001
ESRD	2.20 (1.93–2.52)	1.56 (1.35–1.80)	<0.001	1.79 (1.47–2.18)	1.37 (1.11–1.69)	0.003
Notification year						
2011	Ref	Ref		Ref	Ref	
2012	1.06 (0.96–1.17)	1.03 (0.93–1.14)	0.550	1.12 (0.99–1.27)	1.09 (0.96–1.24)	0.210
2013	1.18 (1.07–1.30)	1.19 (1.08–1.32)	<0.001	1.13 (1.00–1.29)	1.14 (1.00–1.30)	0.050
2014	1.41 (1.28–1.55)	1.42 (1.29–1.57)	<0.001	1.48 (1.31–1.67)	1.47 (1.29–1.67)	<0.001
2015	1.37 (1.24–1.51)	1.37 (1.23–1.51)	<0.001	1.61 (1.43–1.83)	1.53 (1.35–1.74)	<0.001
2016	1.45 (1.32–1.60)	1.38 (1.25–1.53)	<0.001	1.80 (1.60–2.04)	1.57 (1.39–1.79)	<0.001
2017	1.31 (1.19–1.45)	1.15 (1.04–1.28)	0.010	1.63 (1.43–1.84)	1.38 (1.21–1.58)	<0.001

**Note:** Data are presented as numbers (%) or median (IQR)

**Abbreviations:** DM, diabetes mellitus; TB, tuberculosis; AFB, acid-fast bacillus; CCI, and Charlson comorbidity index; HIV, human immunodeficiency virus; ESRD, end-stage renal disease

**Fig 3:**
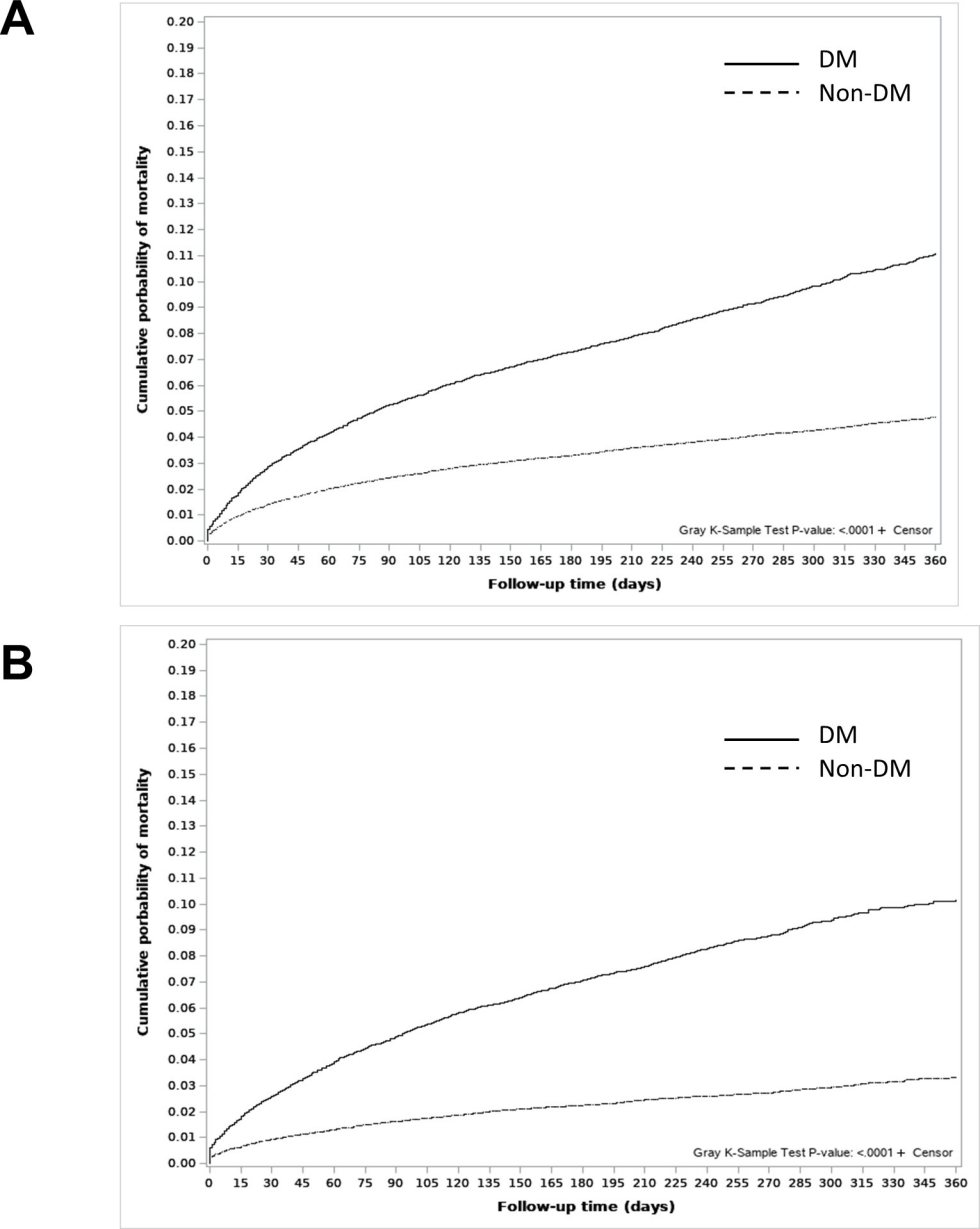
Cumulative mortality curves for non-TB-related deaths in men and women according to DM status. (A) Cumulative mortality curves for non-TB-related deaths in men. (B) Cumulative mortality curves for non-TB-related deaths in women. Abbreviations: TB, tuberculosis; DM, diabetes mellitus.

The cumulative mortality curve for non-TB-related deaths showed a higher probability of deaths in the DM group than in the non-DM group in both men and women (Fig 3, Gray’s test, p<0.001). Moreover, in the multivariate-adjusted sub-distribution hazard model, the unfavorable impact of DM on non-TB-related death was consistent in both men (adjusted hazard ratio, aHR 1.21, 95% CI 1.15–1.27) and women (aHR 1.29, 95% CI 1.20–1.39) (Table 4). Additionally, older age, the lowest household income, and higher CCI scores were unfavorable prognostic factors for non-TB-related deaths in both men and women.

**Table 4:** Impact of DM on non-TB-related deaths in men and women.

Variables	Men (n = 140,939)			Women (n = 98,909)		
	HR	aHR	*p*	HR	aHR	*p*
DM	2.30 (2.19–2.41)	1.21 (1.15–1.27)	<0.001	3.26 (3.06–3.48)	1.29 (1.20–1.39)	<0.001
Age, years						
18–24	Ref	Ref		Ref	Ref	
25–34	1.18 (0.62–2.22)	1.11 (0.59–2.09)	0.750	2.80 (1.18–6.65)	2.76 (1.16–6.56)	0.020
35–44	4.69 (2.71–8.11)	3.87 (2.24–6.69)	<0.001	4.45 (1.91–10.35)	3.93 (1.68–9.16)	0.002
45–54	12.28 (7.23–20.87)	8.32 (4.89–14.15)	<0.001	11.49 (5.08–25.95)	8.54 (3.77–19.39)	<0.001
55–64	24.03 (14.19–40.71)	13.96 (8.23–23.67)	<0.001	19.26 (8.58–43.20)	11.88 (5.26–26.82)	<0.001
65–74	47.19 (27.90–79.83)	24.21 (14.29–41.01)	<0.001	45.89 (20.62–102.14)	27.02 (12.07–60.48)	<0.001
≥ 75	87.32 (51.68–147.54)	43.88 (25.92–74.29)	<0.001	111.05 (50.06–246.36)	62.18 (27.88–138.70)	<0.001
Household income						
0 (Lowest)	1.39 (1.29–1.50)	1.50 (1.38–1.62)	<0.001	1.97 (1.79–2.18)	1.56 (1.41–1.72)	<0.001
1	0.66 (0.61–0.71)	1.10 (1.01–1.19)	0.020	0.81 (0.73–0.90)	1.14 (1.03–1.27)	0.010
2	0.51 (0.46–0.55)	0.98 (0.90–1.07)	0.610	0.60 (0.53–0.67)	1.11 (0.98–1.26)	0.100
3	0.59 (0.55–0.64)	1.04 (0.96–1.12)	0.370	0.64 (0.57–0.71)	1.10 (0.99–1.23)	0.090
4	0.70 (0.65–0.75)	0.98 (0.91–1.05)	0.580	0.71 (0.64–0.79)	1.03 (0.93–1.15)	0.580
5 (Highest)	Ref			Ref	Ref	
TB Lesion						
Pulmonary	Ref	Ref		Ref	Ref	
Extra-pulmonary	1.11 (1.03–1.19)	0.81 (0.74–0.88)	<0.001	0.98 (0.90–1.07)	0.79 (0.71–0.88)	<0.001
Prior TB history						
No	Ref	Ref		Ref	Ref	
Yes	1.08 (1.01–1.15)	0.96 (0.90–1.03)	0.240	0.82 (0.73–0.92)	0.97 (0.86–1.09)	0.600
AFB smear						
Negative	Ref	Ref		Ref	Ref	
Positive	1.04 (0.99–1.15)	1.07 (1.01–1.13)	0.030	1.07 (1.00–1.15)	0.88 (0.81–0.95)	0.002
Unknown	0.82 (0.76–0.89)	0.77 (0.70–0.86)	<0.001	0.75 (0.69–0.83)	0.68 (0.60–0.77)	<0.001
TB culture						
Negative	Ref	Ref		Ref	Ref	
Positive	0.95 (0.90–1.01)	0.84 (0.79–0.90)	<0.001	1.06 (0.97–1.15)	0.88 (0.81–0.95)	0.010
Unknown	1.03 (0.97–1.10)	1.29 (1.19–1.40)	<0.001	1.05 (0.96–1.14)	1.47 (1.31–1.64)	<0.001
CCI score						
0	Ref	Ref		Ref	Ref	
1	2.00 (1.87–2.14)	1.17 (1.10–1.26)	<0.001	1.49 (1.35–1.64)	0.89 (0.81–0.99)	0.030
2	4.58 (4.17–5.03)	2.12 (1.92–2.34)	<0.001	6.01 (5.33–6.78)	2.00 (1.76–2.27)	<0.001
≥3	8.18 (7.66–8.74)	2.63 (2.44–2.83)	<0.001	7.61 (6.93–8.36)	2.10 (1.90–2.33)	<0.001
Co-morbidities						
Transplantation	2.92 (2.34–3.66)	1.40 (1.09–1.81)	0.010	4.03 (2.84–5.70)	2.80 (1.86–4.22)	<0.001
HIV	3.14 (2.38–4.14)	3.41 (2.53–4.60)	<0.001	3.90 (1.47–10.34)	1.54 (0.46–5.17)	0.490
Cancer	7.47 (7.02–7.94)	4.73 (4.41–5.08)	<0.001	5.63 (5.02–6.32)	5.28 (4.59–6.07)	<0.001
ESRD	5.90 (5.43–6.41)	2.87 (2.59–3.17)	<0.001	7.12 (6.37–7.96)	3.72 (3.24–4.28)	<0.001
Notification year						
2011	Ref	Ref		Ref	Ref	
2012	1.24 (1.13–1.37)	1.15 (1.05–1.27)	0.004	1.15 (1.00–1.33)	1.08 (0.93–1.24)	0.320
2013	1.28 (1.16–1.41)	1.18 (1.06–1.30)	0.002	1.40 (1.22–1.61)	1.30 (1.13–1.50)	<0.001
2014	1.58 (1.43–1.74)	1.36 (1.24–1.51)	<0.001	1.62 (1.42–1.86)	1.41 (1.22–1.62)	<0.001
2015	1.85 (1.68–2.03)	1.50 (1.36–1.65)	<0.001	1.90 (1.66–2.17)	1.51 (1.32–1.73)	<0.001
2016	1.95 (1.77–2.14)	1.47 (1.33–1.62)	<0.001	2.14 (1.88–2.44)	1.52 (1.33–1.75)	<0.001
2017	2.19 (2.00–2.40)	1.52 (1.37–1.67)	<0.001	2.56 (2.25–2.91)	1.75 (1.53–2.01)	<0.001

**Note:** Data are presented as numbers (%) or median (IQR)

**Abbreviations:** DM, diabetes mellitus; TB, tuberculosis; AFB, acid-fast bacillus; CCI, and Charlson comorbidity index; HIV, human immunodeficiency virus; ESRD, end-stage renal disease

## Discussion

Through an analysis of a nationwide integrated TB cohort, we found that DM had an unfavorable effect on mortality during TB treatment. Regarding TB-related deaths, DM showed a negative effect in men but not in women. For non-TB-related deaths, the negative effect of DM was consistent in both men and women.

Previous studies [11–16] have reported that DM increases the risk of poor TB treatment outcomes. DM is associated with delayed culture conversion, which could lead to treatment failure [17, 18]. Although DM is a well-known poor prognostic factor in patients with TB, the mechanism by which it increases mortality in those with TB remains unclear. Several hypotheses exist regarding the increased TB-related mortality in patients with DM. One hypothesis is that hyperglycemia impairs innate and adaptive immune responses, rendering patients more susceptible to infections, including TB [22]. Studies involving animal models and human plasma cells have shown alterations in cytokine responses, including T-helper (Th) 1, Th 2, Th 17, and interferon-gamma, in chronic hyperglycemic conditions [23–27]. Thus, the capacity for microbiological control could be reduced, leading to severe TB at the time of diagnosis, microbiological failure, and poor treatment outcomes, ultimately contributing to increased mortality. Another explanation for the poor treatment outcomes is the different pharmacokinetic and pharmacodynamic effects of anti-TB drugs in patients with DM. Babalik et al. found decreased isoniazid and rifampicin concentrations in patients with DM [28]. The authors suggested that decreased gastrointestinal absorption or increased distribution of isoniazid and rifampicin in patients with DM could result in a decline in their concentrations [28]. This decreased drug concentration could result in poor pharmacologic control of mycobacteria and unfavorable treatment outcomes. Additionally, higher mortality in patients with DM could result from non-microbiological factors, such as hemoptysis and combined pneumonia. Patients with DM and TB tended to have more severe clinical manifestations, such as severe lung involvement with cavitation and positive AFB smear [29–31], than patients with TB alone. Moreover, this severe form of TB can predispose the individual to pulmonary complications, including hemoptysis and additional infection.

In our study, TB was the most common cause of death (47.9%); however, non-TB-related deaths were also substantial (52.1%) during TB treatment and were more common in the older population. TB-related and non-TB-related deaths were higher in the DM group than in the non-DM group; however, the difference between the two groups was more notable for non-TB-related deaths in the survival curve. Patients with TB and DM were older and had more co-morbidities than patients with TB alone. The TANDME Study group showed a higher risk of cardiovascular disease in patients with TB-DM than in non-DM-TB patients [30]. Active inflammation caused by TB can aggravate chronic co-morbid conditions, such as ischemic heart disease, heart failure, and chronic respiratory disease [32–35]. Thus, TB could have contributed to the higher rate of non-TB-related death during treatment in the DM group. Our results highlight the increasing role of DM in TB control. The End TB Strategy emphasizes the importance of coordinated clinical care when TB is newly diagnosed in patients with DM [36]. A further integrated approach is needed to optimize treatment for both TB and DM. It will benefit patient outcomes if clinicians treat patients with DM-presumed TB early when TB-related symptoms occur. Screening patients with TB for DM would also contribute to improved TB treatment outcomes.

Notably, the negative impact of DM on non-TB-related deaths was consistent in both men and women; however, the impact on TB-related deaths was more prominent in men in our study. Considering that the median age of women was higher than that of men, the excessive impact of age may reduce the effects of other confounding factors, including DM. It is also possible to consider that other confounding factors, such as smoking and alcohol consumption, may negatively impact TB treatment outcomes in men with DM. However, additional analysis of these factors was not performed in this study because of a lack of information.

The major strength of our study is that, first, this is a nationwide cohort study to investigate the impact of DM on mortality in patients with TB. This cohort covered almost all registered patients with TB and had adequate follow-up periods. Second, we could analyze and adjust more relevant covariates including socioeconomic status and multiple co-morbidities, which are crucial contributing factors to the mortality of patients with TB and DM, by integrating three different national datasets. Finally, to our best knowledge, this is the first study to separately investigate the impact of DM on TB-related and non-TB-related deaths. Dividing mortality into TB-related and non-TB-related factors has helped in-depth consideration of mortality reduction strategies in TB-DM co-prevalent patients in national TB programs. Despite these strengths, this study has some limitations. We were unable to access certain information owing to the retrospective nature of the study design. Due to the limitations of the integrated database, we could not analyze some confounding variables such as HbA1c, smoking, drinking, body mass index, and dietary intake. To overcome these limitations, we aimed to adjust for the available information as possible, including comorbidities and the CCI. In particular, we included CCI in the multivariate analysis, which covers cardiovascular diseases, renal diseases, and neurological disorders that can develop as complications of DM.

In conclusion, DM is independently associated with a greater hazard of death among TB patients during the treatment for both TB-related death and non-TB-related death. Our study suggests that screening for and providing appropriate treatment for DM should be considered among patients with TB. Exploring the optimal integrated clinical approach for both TB and DM could be valuable for enhancing the treatment outcomes.

## Supporting information

S1 Fig Number of TB-related and non-TB-related deaths by time after treatment initiation. (A) Number of TB-related and non-TB-related deaths by time after treatment initiation in the DM group. (B) Number of TB-related and non-TB-related deaths by time after treatment initiation in the non-DM group. Abbreviations: TB, tuberculosis; DM, diabetes mellitus.
(ZIP)

S2 Fig Top 10 causes of death for non-TB-related deaths. (A) Top 10 causes of non-TB-related deaths in the DM group. (B) Top 10 causes of non-TB-related deaths in the non-DM group. Abbreviations: TB, tuberculosis; DM, diabetes mellitus.
(ZIP)
