# Supplementary figures and images for: Association between diabetes mellitus and cause of death in patients with tuberculosis: A Korean nationwide cohort study
Source: PLoS One. 2023 Dec 14;18(12):e0295556. doi: 10.1371/journal.pone.0295556 (PMC10721100; doi:10.1371/journal.pone.0295556)

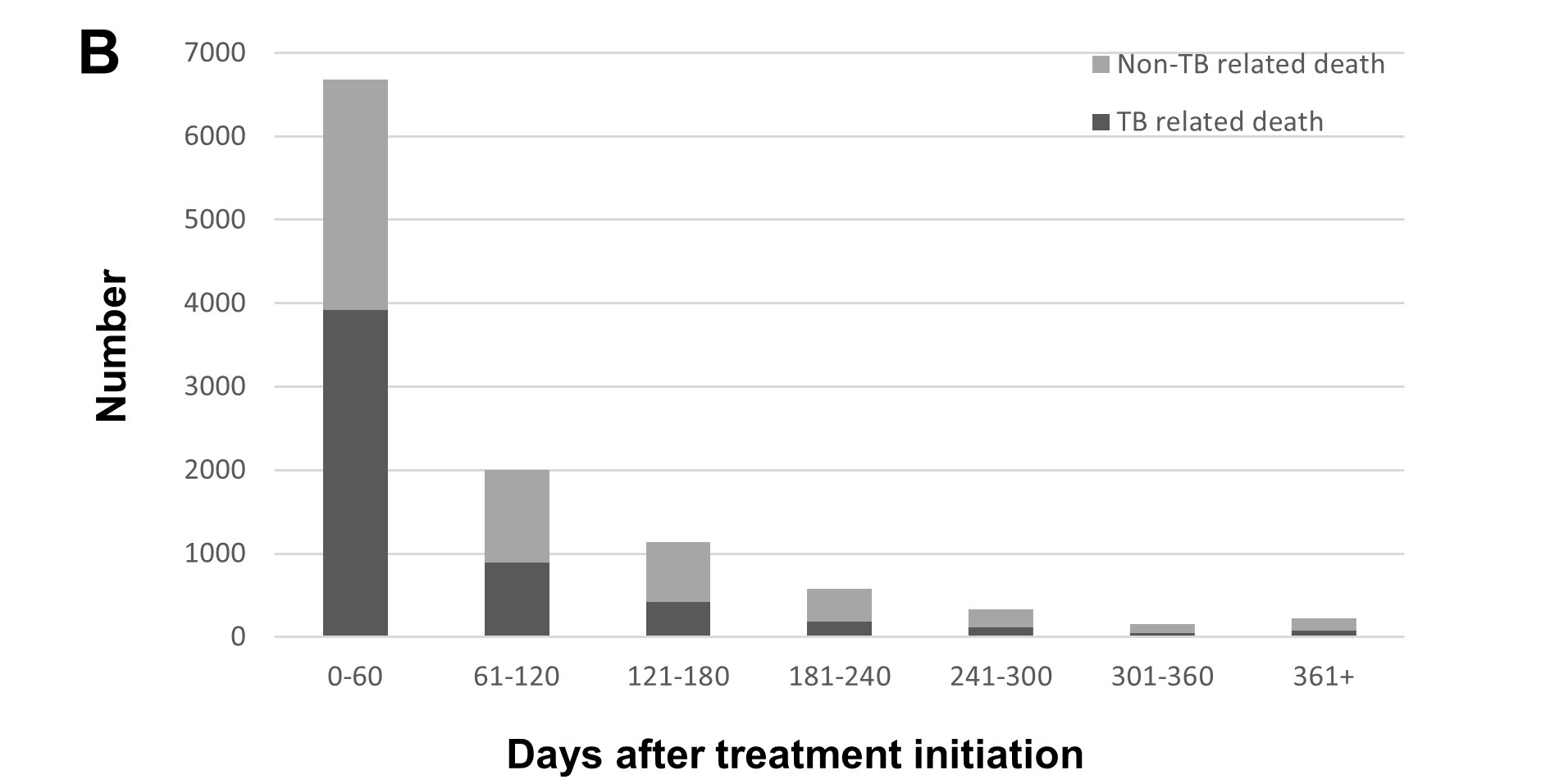

Supplement: S1 Fig — (A) Number of TB-related and non-TB-related deaths by time after treatment initiation in the DM group. (B) Number of TB-related and non-TB-related deaths by time after treatment initiation in the non-DM group. Abbreviations: TB, tuberculosis; DM, diabetes mellitus. (ZIP) [file pone.0295556.s001.zip › Supple Fig1(B).tif]

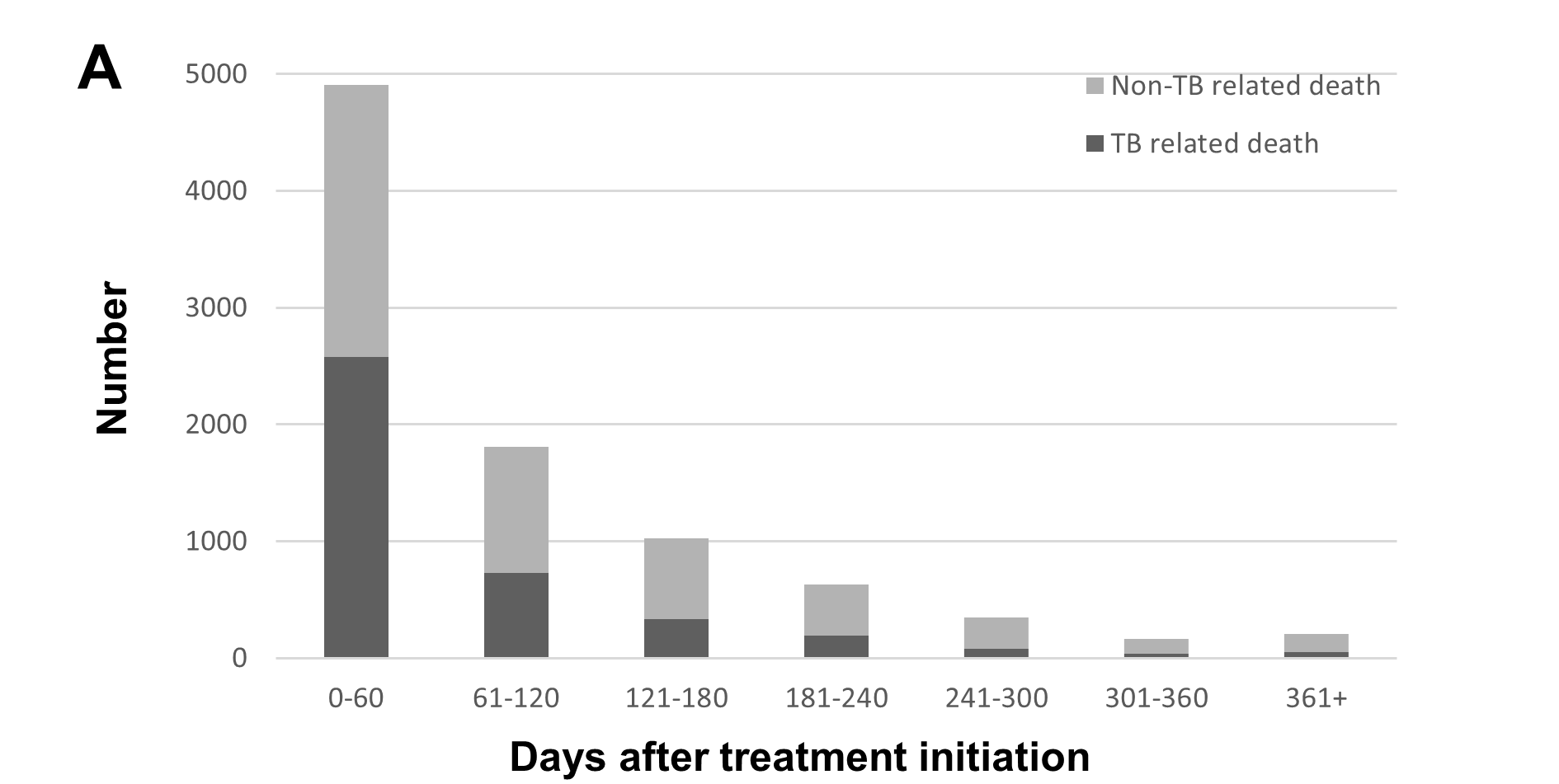

Supplement: S1 Fig — (A) Number of TB-related and non-TB-related deaths by time after treatment initiation in the DM group. (B) Number of TB-related and non-TB-related deaths by time after treatment initiation in the non-DM group. Abbreviations: TB, tuberculosis; DM, diabetes mellitus. (ZIP) [file pone.0295556.s001.zip › Supple Fig1(A).tif]

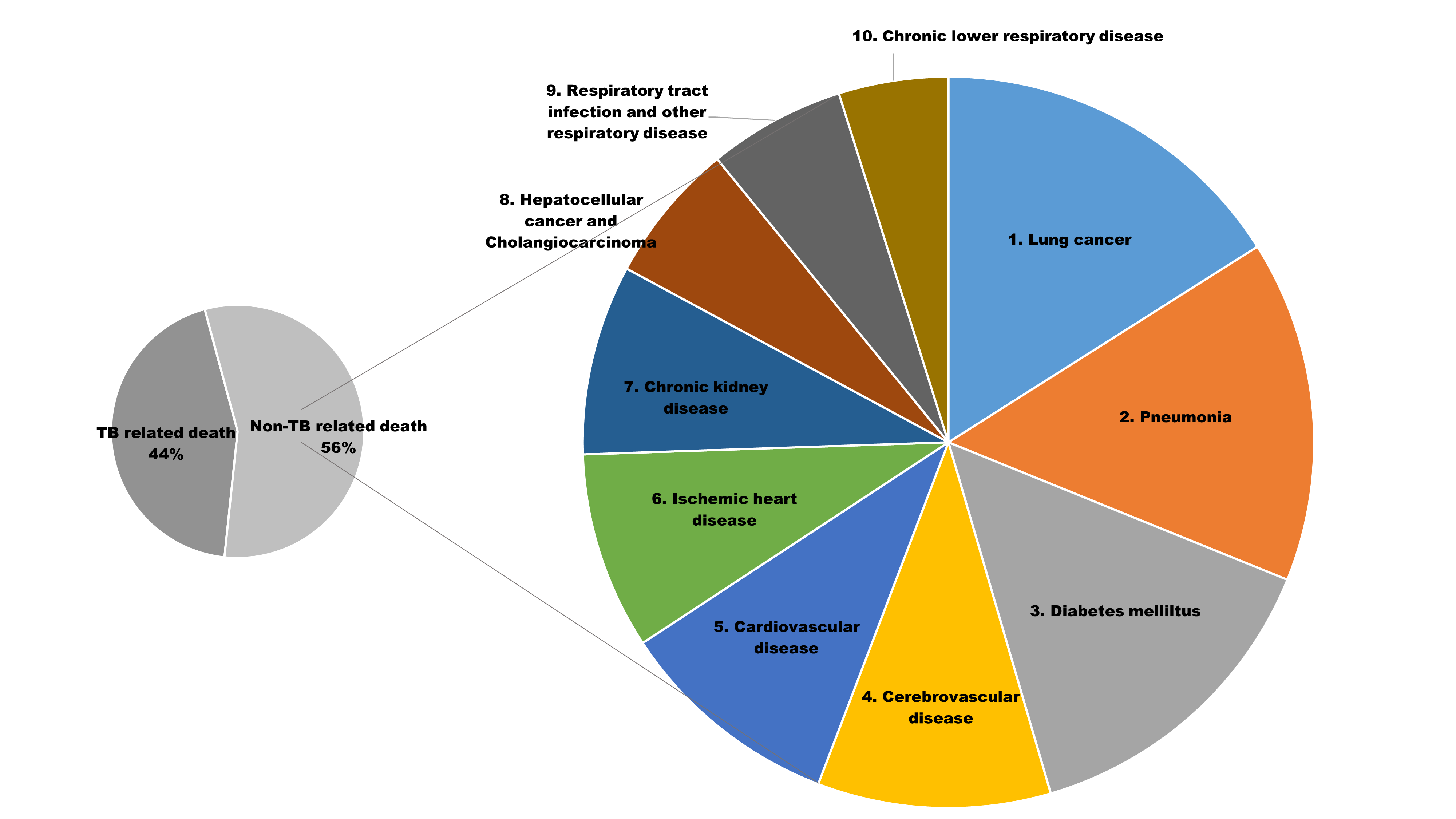

Supplement: S2 Fig — (A) Top 10 causes of non-TB-related deaths in the DM group. (B) Top 10 causes of non-TB-related deaths in the non-DM group. Abbreviations: TB, tuberculosis; DM, diabetes mellitus. (ZIP) [file pone.0295556.s002.zip › Supple Fig2(A).TIF]

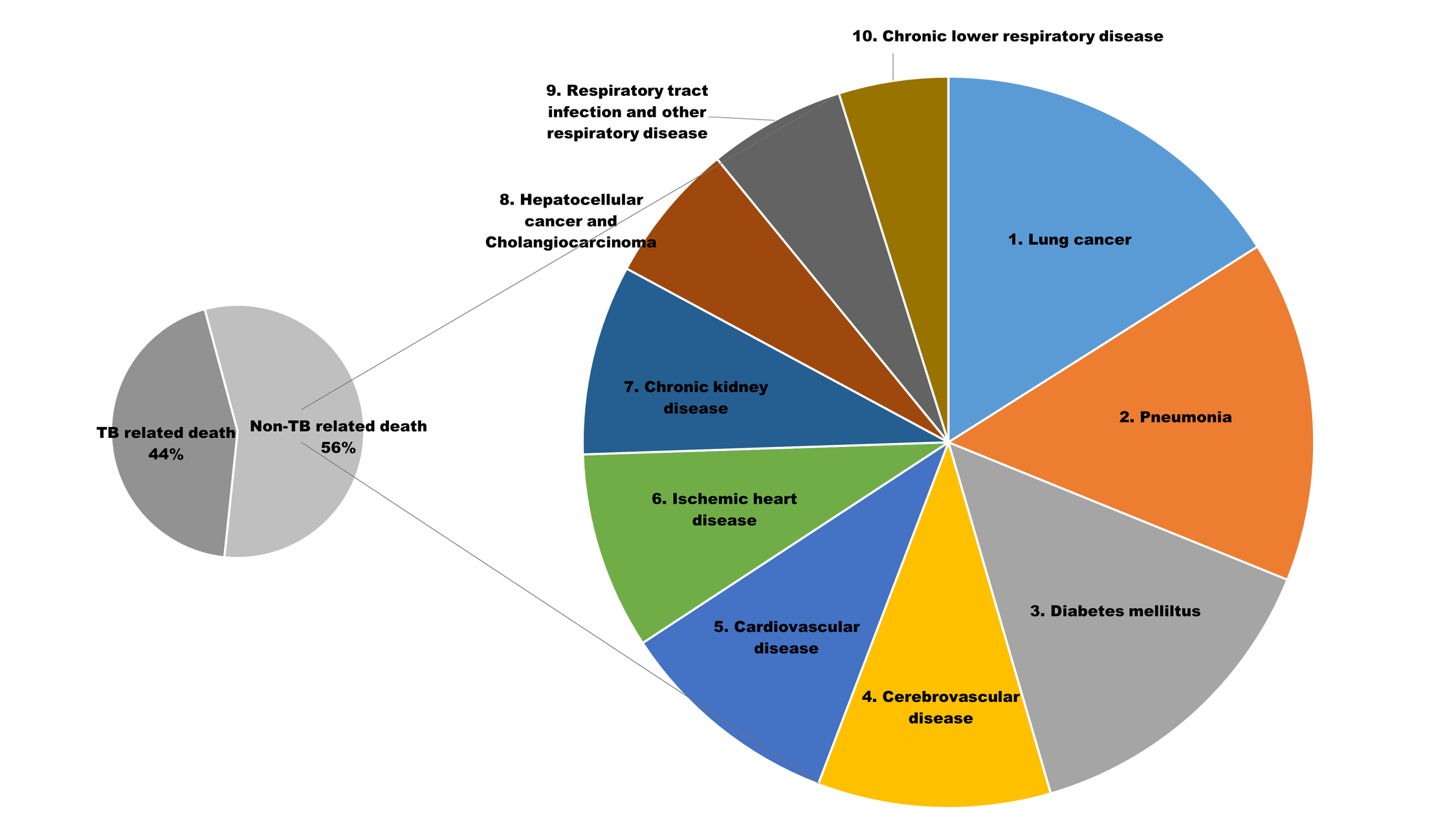

Supplement: S2 Fig — (A) Top 10 causes of non-TB-related deaths in the DM group. (B) Top 10 causes of non-TB-related deaths in the non-DM group. Abbreviations: TB, tuberculosis; DM, diabetes mellitus. (ZIP) [file pone.0295556.s002.zip › Supple Fig2(A) (1).tif]
